# Live-cell imaging reveals square shape spindles and long mitosis duration in the silkworm holocentric cells

**DOI:** 10.17912/micropub.biology.000441

**Published:** 2021-09-01

**Authors:** Lucien Vanpoperinghe, Frederique Carlier-Grynkorn, Gaetan Cornilleau, Takahiro Kusakabe, Ines A Drinnenberg, Phong T Tran

**Affiliations:** 1 Institut Curie, PSL Université, Sorbonne Université, CNRS, Paris, France; 2 Médicine Sorbonne Université, École Normale Supérieure, Paris, France; 3 Kyushu University, Department of Bioresource Sciences, Laboratory of Insect Genome Science, Fukuoka, Japan; 4 University of Pennsylvania, Department of Cell and Developmental Biology, Philadephia, PA, United States

## Abstract

Proper chromosome segregation during mitosis requires both the assembly of a microtubule (MT)-based spindle and the assembly of DNA-centromere-based kinetochore structure. Kinetochore-to-MT attachment enables chromosome separation. Monocentric cells, such as found in human, have one unique kinetochore per chromosome. Holocentric cells, such as found in the silkworm, in contrast, have multiple kinetochore structures per chromosome. Interestingly, some human cancer chromosomes contain more than one kinetochore, a condition called di- and tricentric. Thus, comparing how wild-type mono- and holocentric cells perform mitosis may provide novel insights into cancer di- and tricentric cell mitosis. We present here live-cell imaging of human RPE1 and silkworm BmN4 cells, revealing striking differences in spindle architecture and dynamics, and highlighting differential kinesin function between mono- and holocentric cells.

**Figure 1. Holocentric BmN4 cells have “square” spindle architecture and long metaphase duration.**
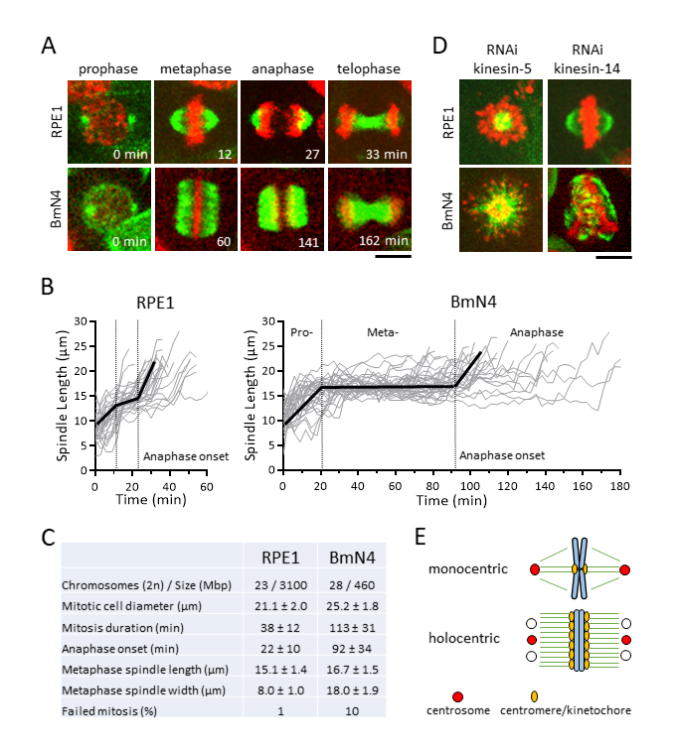
A. Time-lapse images of human hTERT-RPE1 and silkworm Bmn4-SID1 cells undergoing mitosis (green = microtubules; red = DNA). Scale bar, 10 μm. B. Spindle length versus time plot for RPE1 (n = 27 cells) and BmN4 (n = 31 cells) during mitosis (bold dark lines mark average spindle length; dashed lines mark transition of mitotic phases). C. Mitotic spindle parameters of wild-type RPE1 and BmN4 cells (mean ± standard deviation). D. Metaphase spindle phenotypes of RPE1 and BmN4 cells RNAi-mediated depletion of kinesin-5 and kinesin-14. Scale bar, 10 μm. E. Cartoon model of monocentric and holocentric spindle and chromosome architecture – square spindle may have multiple centrosome-like microtubule organizing centers.

## Description

Human hTERT-RPE1 cells represent wild-type monocentric cells as they were immortalized, via telomerase expression, from normal somatic retinal pigment epithelium cells (Jiang *et al.* 1999). Silkworm BmN4-SID1 cells represent wild-type holocentric cells as they were spontaneously immortalized from somatic ovary cells, and modified with the SID1 gene from *C. elegans* for RNAi-mediated depletion (Mon *et al.* 2012). Interestingly, cancer such as leukemia and lymphoma often exhibits dicentric or tricentric chromosomes (Herry *et al.* 2007; M’Kacher *et al.* 2020), a condition reminiscent of holocentric cells. Further, monocentric cells induced to be dicentric showed transformed cancerous properties (Gascoigne and Cheeseman 2013). We seek to understand the differences in spindle assembly dynamics and chromosome segregation in wild-type monocentric RPE1 and holocentric BmN4 cells, to potentially reveal mechanisms of how di- or tricentric cancer cells divide.

We first established protocols for optimal live-cell imaging of RPE1 and BmN4 cells undergoing mitosis (see Methods). Using the vital dyes SPY-tubulin and SPY-DNA to label cells, and the spinning disk confocal microscope, we imaged RPE1 cells for 3 hr and BmN4 cells for 9 hr with no discernible photo damage. In comparing mitosis between RPE1 and BmN4 cells, striking differences in spindle dynamics and spindle architecture were observed. The RPE1 spindles exhibited the canonical mitotic phase transition (Fig. 1A). At prophase we observed the initial assembly of the bipolar spindle from two foci, likely the centrosome; at metaphase we observed the stable-length spindle with compacted metaphase plate-aligned chromosomes and the canonical “oval” shape spindle, with an aspect ratio of ~2 (15.1 μm length/8.0 μm width, Fig. 1C); at anaphase we observed chromosome separation concurrent with spindle elongation; at telophase we observed the compacted microtubule midzone, the future midbody (Fig. 1A). Total mitosis duration, from prophase to telophase, in RPE1 cells was ~38 min, with the onset of anaphase at ~22 min (Fig. 1B, 1C). In contrast, BmN4 spindles also exhibited clear mitotic phase transition, but with distinct differences. At prophase we mostly observed the assembly of the bipolar spindle from two foci, but in ~14% of cells spindle microtubules nucleated from more than two foci, with small microtubules present everywhere near the chromosomes, indicating potential centrosomal and acentrosomal MT nucleation (Mon *et al.* 2014); at metaphase we observed the stable-length spindle with compacted metaphase plate-aligned chromosomes and striking “square” shape spindle, with an aspect ratio of ~1 (16.7 μm length/18.0 μm width, Fig. 1C), and no clear foci indicative of spindle poles; at anaphase we observed chromosome separation concurrent with spindle elongation; at telophase we observed the compacted microtubule midzone (Fig. 1A). The square spindle of BmN4 cells is reminiscent of the square spindle of plant cells, which are also holocentric (de Keijzer *et al.* 2014; Zhang and Dawe 2011). Total mitosis duration in BmN4 cells was ~113 min, with the onset of anaphase at ~92 min (Fig. 1B, 1C). BmN4 cells took ~4X longer time to complete mitosis, with corresponding ~4X longer time spent in metaphase, compare to RPE1 cells (Fig. 1C). We also observed that mitotic failure, defined as spontaneous cell death or aneuploid cell division, occurred in ~1% (1 of 79 spindles) of RPE1 cells, compared to ~10% (22 of 207 spindles) of BmN4 cells (Fig. 1C). Given that RPE1 and BmN4 cells have similar chromosome number and round mitotic cell size (Fig. 1C), we propose that holocentricity, where multiple kinetochores are present on one chromosome, requires longer time to make proper kinetochore-to-MT attachment at metaphase as compared to monocentricity. An exception maybe at embryogenesis or early development, where mitosis occurs relatively fast, compared to adult somatic cells such as RPE1 and BmN4. The extended metaphase duration and higher frequency of failed mitosis may be a consequence of the unusual “square” spindle architecture, or of the multiple kinetochores per chromosome.

Spindle assembly dynamic is orchestrated by microtubule-associated proteins, many of which are kinesins (Mountain and Compton 2000). In human cells, kinesin-5 Kif11 is essential for bipolar spindle assembly and elongation, as its inhibition results in monopolar spindle and subsequent cell death (Mayer *et al.* 1999; Sawin *et al.* 1992); and Kinesin-14 HSET is required to focus the microtubule minus ends at the spindle poles, and its inhibition, while having little effect on the metaphase spindle in normal cells, causes catastrophic multipolar spindle defects in cancer cells with supernumerary centrosomes (Kleylein-Sohn *et al.* 2012; Kwon *et al.* 2008). To compare the function of kinesin-5 and -14 in RPE1 and BmN4 cells, we performed RNAi-mediated depletion. Consistent with previous reports, we observed monopolar spindles in kinesins-5 siRNA-treated RPE1 cells, and seemingly normal metaphase spindles in kinesin-14 siRNA-treated RPE1 cells (Fig. 1D). In contrast, while we also observed monopolar spindles in kinesins-5 dsRNA-treated BmN4 cells, we observed mal-shaped metaphase spindles and unaligned chromosomes in kinesins-14 dsRNA-treated BmN4 cells (Fig. 1D). The result indicates that BmN4 cells need kinesin-14 for proper spindle assembly and chromosome segregation, more so than RPE1 cells, and highlights the importance of differential motor function in the context of mono- and holocentricity.

In summary, the holocentric silkworm cell BmN4 is an emergent model system to investigate novel pathways of spindle assembly and centromere-kinetochore assembly (Cortes-Silva *et al.* 2020; Li *et al.* 2019; Mon *et al.* 2014; Mon *et al.* 2017; Senaratne *et al.* 2021). We define similarities and differences in spindle architecture and dynamics between BmN4 and monocentric human RPE1 cells (Fig. 1E). Future work should probe the impact of the centrosome, the centromere/kinetochore, the spindle assembly checkpoint, on spindle architecture and dynamics and chromosome segregation. This work sets the stage for pinpointing and investigating specific genes/proteins affecting only BmN4 cells, but not RPE1 cells, in mono- and tricentric human cancer cells.

## Methods

**Cells**

We used human hTERT-RPE1 (RRID:CVCL_4388) and silkworm BmN4-SID1 (RRID:CVCL_Z091) cells (Bodnar *et al.* 1998; Mon *et al.* 2012). These immortalized cells are considered normal diploid cells, as they have proper number of chromosomes and do not exhibit transformed phenotypes. An added benefit is that they are easily amenable to RNAi-mediated depletion, enabling functional studies of protein-of-interest. Cell culture condition is well-established (Bodnar *et al.* 1998; Mon *et al.* 2012). Briefly, hTERT-RPE1 cells were maintained in DMEM/F-12 medium (GIBCO Cat# 21041-025) supplemented with 10% fetal calf serum (Biowest Cat# S181T-500), 1% penicillin-streptomycin (GIBCO Cat# 15140-22) in 5% CO_2_ incubator at 37°C; and BmN4-SID1 cells were maintained in Sf-900 II SFM medium (GIBCO Cat# 10902-088) supplemented with 5% fetal calf serum (Biowest Cat# S181T-500), 1% penicillin-streptomycin (GIBCO Cat# 15140-122) and 1% L-glutamine (GIBCO Cat# 25030-024) in a 27°C incubator.

**RNAi**

RNA-interference is well-established for RPE1 cells (Jackson *et al.* 2006). Briefly, we treated RPE1 cells with 20 nM of siRNA-mediated kinesin-5 or kinesin-14 depletion reagents (Dharmacon ON-TARGETplus siRNA SMARTPool) 48 hr prior to imaging. A subset of the treated-cells were used for RT-qPCR to determine the level of mRNA depletion. RNA-interference for BmN4 cells has been established (Mon *et al.* 2012), utilizing the ability of BmN4-SID1 cells to soak up dsRNA in the medium to perform RNA-interference. Briefly, ~300 bp of dsRNA targeting silkworm kinesin-5 and kinesin-14 were generated from DNA templates flanked by T7 promoter sequences on both sides using the MAXIscript T7 Transcription Kit (Thermo Fisher Scientific, Cat# AM1312) . We treated BmN4-SID1 cells with 20 nM of dsRNA 48 hr prior to imaging. A subset of the treated-cells were used for RT-qPCR to determine the level of mRNA depletion. The cells were cultured in a 2 mL FluoroDish (World Precision Instruments, Cat# FD35-100).

**Live-cell imaging**

One hour prior to imaging, SPY-DNA-555nm (Spirochrome Cat# SC201) and SPY-tubulin-650nm (Spirochrome Cat# SC503) dyes were added to the medium at a 10,000X dilution. Cells were imaged using the spinning disk confocal microscope. Briefly, the Nikon Eclipse Ti-E perfect focus inverted microscope, with 40X/1.3 N.A. Plan Apo oil immersion objective lens and Mad City Piezo stepper stage, coupled to the Yokogawa CSU-X1 spinning disk confocal unit, the Photometrics Cascade EM-CCD camera, the Gataca Systems laser unit with 561 nm (100 mW) and 642 nm (100 mW) lines, controlled by Molecular Devices software MetaMorph 7.8, enclosed within a thermal box to keep stable temperatures of 27°C (BmN4 cells) or 37°C (RPE1 cells). Movies were made with the following parameters: laser power 5mW, EM-gain 300, Bin 1X, exposure time 100-200 ms, 13 optical z-sections, 2 µm spacing per 3D stack, 3 min time interval between stacks, and 3 hr movies (RPE1 cells) or 9 hr movies (BmN4 cells).

## Reagents

Dharmacon ON-TARGETplus siRNA SMARTPool Cat# L-003317-00-0005 (human kinesin-5 KIF11)

Dharmacon ON-TARGETplus siRNA SMARTPool Cat# L-004958-00-0005 (human kinesin-14 KIFC1)

*Bombyx mori* primers to generate T7 template for kinesin-5 dsRNA generation:

sense: GCTGGCTACAATTGCACTGT

antisense: TCATGAGTGTCGAAGCCACT

*Bombyx mori* primers to generate T7 template for kinesin-14 dsRNA generation:

sense: TGCTGCACCTCGGATTAAGA

antisense: CCTCTAGAAGCTTTGTCTTACGT
